# Fine Mapping and Candidate Gene Analysis of Rice Grain Length QTL *qGL9.1*

**DOI:** 10.3390/ijms241411447

**Published:** 2023-07-14

**Authors:** Luomiao Yang, Peng Li, Jingguo Wang, Hualong Liu, Hongliang Zheng, Wei Xin, Detang Zou

**Affiliations:** Key Laboratory of Germplasm Enhancement, Physiology and Ecology of Food Crops in Cold Region, Ministry of Education, Northeast Agricultural University, Harbin 150030, China

**Keywords:** *Oryza sativa* L., grain length, re-sequencing, fine mapping, P450 protein

## Abstract

Grain length (GL) is one of the crucial determinants of rice yield and quality. However, there is still a shortage of knowledge on the major genes controlling the inheritance of GL in *japonica* rice, which severely limits the improvement of japonica rice yields. Here, we systemically measured the GL of 667 F_2_ and 1570 BC_3_F_3_ individuals derived from two cultivated rice cultivars, Pin20 and Songjing15, in order to identify the major genomic regions associated with GL. A novel major QTL, *qGL9.1*, was mapped on chromosome 9, which is associated with the GL, using whole-genome re-sequencing with bulked segregant analysis. Local QTL linkage analysis with F_2_ and fine mapping with the recombinant plant revealed a 93-kb core region on *qGL9.1* encoding 15 protein-coding genes. Only the expression level of *LOC_Os09g26970* was significantly different between the two parents at different stages of grain development. Moreover, haplotype analysis revealed that the alleles of Pin20 contribute to the optimal GL (9.36 mm) and GL/W (3.31), suggesting that Pin20 is a cultivated species carrying the optimal GL variation of *LOC_Os09g26970*. Furthermore, a functional-type mutation (16398989-bp, G>A) located on an exon of *LOC_Os09g26970* could be used as a molecular marker to distinguish between long and short grains. Our experiments identified *LOC_Os09g26970* as a novel gene associated with GL in *japonica* rice. This result is expected to further the exploration of the genetic mechanism of rice GL and improve GL in rice *japonica* varieties by marker-assisted selection.

## 1. Introduction

Grain size, a complex quantitative trait involving grain length (GL), grain width (GW), grain thickness, and the grain length/width ratio (GL/W), is one of the determinants of grain weight, which not only affects the yield, but also the appearance quality of rice [1,2]. As an important factor affecting rice yield and quality, mining grain-shape-related genes is an important means to understanding their molecular mechanism and genetic basis. As of 2023, at least 201 rice grain shape genes have been identified. They are located on all chromosomes of the rice genome, and most are distributed on chromosomes 1, 2, 5, 6, and 7 (https://pubmed.ncbi.nlm.nih.gov/) (accessed on 7 May 2023). Most of these 201 genes directly regulate the rice grain shape, and the remaining genes indirectly regulate the grain size through an interaction network between genes. 

Previous studies have found that the major factors affecting grain size include the ubiquitination-protease pathway, G-protein signaling, mitogen-activated protein kinase signaling, phytohormone regulation, and various transcriptional regulators [3]. GRAIN WIDTH 2 (*GW2*), the first QTL cloned in rice, encodes a RING-type E3 ubiquitin ligase with ubiquitination and autoubiquitination activity located in the cytoplasm and nucleus [4]; G-proteins that regulate grain size in rice include the a-subunit encoded by *RGA1/D1* [5]; and the β-subunit encoded by *DEP1* [6]. In addition, synthetic-hormone-related genes, such as *OsTAR1* [7] and *TGW6* [8], are also involved in the regulation of seed size. Moreover, several other transcriptional regulatory modules, such as *OsmiR396-OsGRFs* [9] and *AP2/ERF* modules [10], also play a key role in rice grain shape determination. In sum, rice grain shape is regulated by multiple factors. Even so, the current molecular network of rice grain types is still insufficient to explain all of the genetic variations. It is of great importance to explore new major genes and allelic variations for the high-yield breeding of rice.

It is well known that the grain length of *indica* rice is longer than that of *japonica* rice. Therefore, the excellent allelic variations of some important GL genes cloned at present are from *indica* rice. For example, the loss-of-function variations of *GS3* [11] and *TGW6* [8] are mostly from *indica* rice. *GS5* is a large grain allelic variation that was retained during the domestication of *indica* rice [12]. The overexpression of *LG3* [13] and *GLW7* [14] in *indica* rice can increase the grain length. Using these allelic variations to improve the grain shape of *japonica* rice, in addition to the method of breeding offspring through *indicia–japonica* hybridization, mutants can be directly obtained through gene editing. However, from the perspective of the geographical adaptability of subspecies, when using the offspring of *indica–japonica* hybridization, it is difficult to obtain materials with an excellent background of *japonica* rice, therefore, it is difficult to produce and apply unless it is included in a large number of molecular breeding works. The materials obtained by gene editing also cannot be used as cultivated varieties due to a certain degree of growth defects [15]. Considering this comprehensively, it is wise and efficient to clone new grain shape genes from *japonica* rice and apply them to *japonica* rice breeding.

Heilongjiang Province, as the main production area of early maturing *japonica* rice in China, had a planting area of about 6.43 million hectares in 2022, accounting for more than 15.5% of the national rice area. However, the grain length value of *japonica* rice in this region is low compared to *indica* rice in southern China, which hinders yield improvement. Therefore, the identification of new alleles controlling grain length from local sources is significant to increasing rice yield. Recent efforts combining QTL-seq and linkage analysis have led to the localization of several candidate genes in rice [16,17,18,19]. In this study, we used two *japonica* rice varieties, Pin20 and Songjing 15 (SJ15), with significant differences in grain shape, as parents in order to develop the F_2:3_ population and the BC_3_F_3_ population. Through QTL-seq, linkage analysis, and fine mapping strategies, we identified the long-grain genes in the large-grain-variety of Pin20. Furthermore, the KASP molecular markers were developed to identify plants with different grain types. This will facilitate the in-depth study of grain type improvement and regulatory mechanisms in *japonica* rice varieties.

## 2. Results

### 2.1. Screening and Evaluation of Plant Height

**Figure 1:**
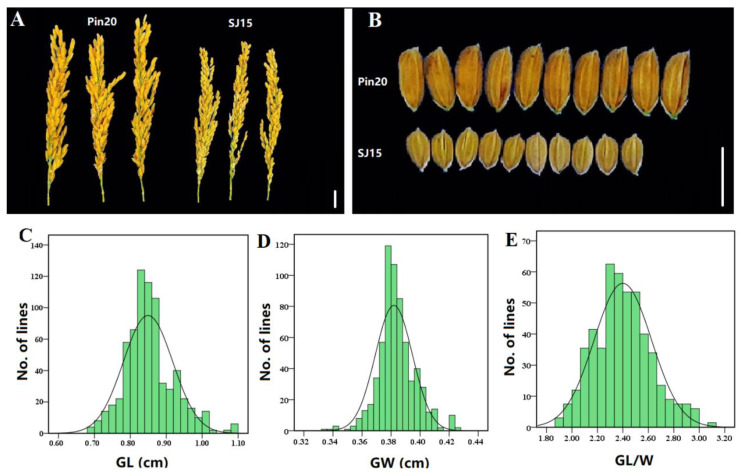
Phenotypic characteristics of grain shape of the parents and F_2:3_ population. (**A**) Performance of the mature spikes of the two parents; (**B**) Comparison of the mature grain types of the two parents; and (**C**–**E**) Frequency distribution of grain length (GL), grain width (GW), and length–width ratio (GL/W) of rice grains in the F_2:3_ population. The scale bars for (**A**,**B**) were 1 cm.

The phenotypic detection showed that there were significant differences in the grain length (GL) and length–width ratio (GL/W) between SJ 15 and Pin 20 (Table 1, Figure 1A,B). For the F_2:3_ population, the variation ranges of GL, grain width (GW), and GL/W were 0.68–1.09 cm, 0.33–0.43 cm, and 1.88–3.09 cm (Table 1, Figure 1C–E), respectively. Except for grain width, the absolute values of the skewness and kurtosis of GL and GL/W were less than 1 (Table 1), showing continuous variation and normal distribution, indicating that these two traits conform to the genetic model of quantitative traits.

### 2.2. Phenotypic Analysis of Extreme DNA Pools of Grain Type

**Figure 2:**
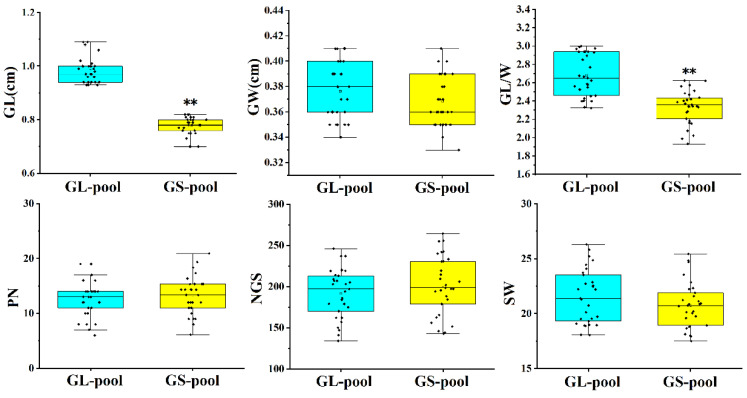
Box plot of panicle traits of individuals with significant differences in grain length. GL-pool, long-grain DNA pool; GS-pool, short-grain DNA pool; GL, grain length; GW, grain width; GL/W, length–width ratio; PN, spike number; NGS, number of grains per spike; and SW, spike weight per plant. ** indicates the significant difference detected at *p* < 0.01 level. Each black dot on the box plot represents the phenotypic value corresponding to a single independent plant.

The differences in individual traits had an impact on the genotype frequency analysis of the DNA hybrid pool on the whole genome. To clarify the differences in the genetic background between the two DNA pools, 30 long-grain and 30 short-grain lines were analyzed for GL, GW, GL/W, spike number (PN), number of grains per spike (NGS), and spike weight per plant (SW). The results showed that there were highly significant differences in GL and GL/W between the long-grain and short-grain pools, while there were no significant differences in GW, PN, NGS, and SW (Figure 2). Therefore, the phenotypic differences between these two DNA mixing pools are distributed only in the GL and GL/W; thus, we selected 30 representative long-grain individuals and 30 short-grain individuals to prepare the GL-pool and GS-pool in order to map the candidate genomic loci using bulked segregant analysis (BSA) and re-sequencing analyses, respectively.

### 2.3. Identification of a Major QTL Controlling GL in Rice Using QTL-Seq

**Figure 3:**
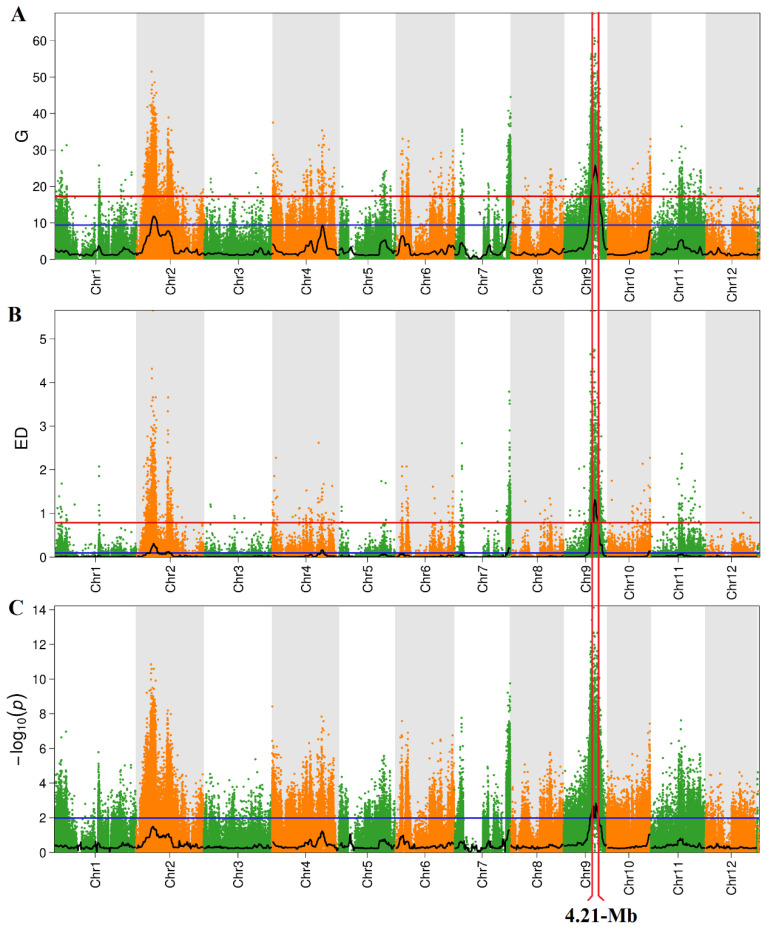
The results of the QTL-Seq analysis. (**A**) The G-statistic value to map *qGL9.1* based on SNP. (**B**) The Euclidean distance algorithm to map *qGL9.1*. (**C**) The two-tailed Fisher’s exact test to map *qGL9.1* based on Indel. The blue lines and the red line represent the threshold line, with confidence levels of 0.95 and 0.99, respectively. The number on the horizontal coordinate represents the chromosome number. The red wireframe represents the intervals covered by *qGL9.1* in different computational modes.

A total of 315,277,920 clean reads and 47,291,688,000 bases were obtained by re-sequencing and data quality control of the two DNA mix pools and both parents (Supplementary Table S1). In addition, the ED (Euclidean distance) and two-tailed Fisher’s exact test for each bulk were calculated by aligning the sequence with the Nipponbare reference genome. After calculating a statistical confidence interval of *p* < 0.01 between the two extreme phenotypic blocks, a 4.21 Mb (14,240,001 bp–18,445,701 bp) genomic region on chromosome 9 was identified by overlapping the results of the three algorithms (Table 2, Figure 3). We designated this QTL as *qGL9.1*.

### 2.4. Narrowing of qGL9.1 to a Fine Region

**Figure 4:**
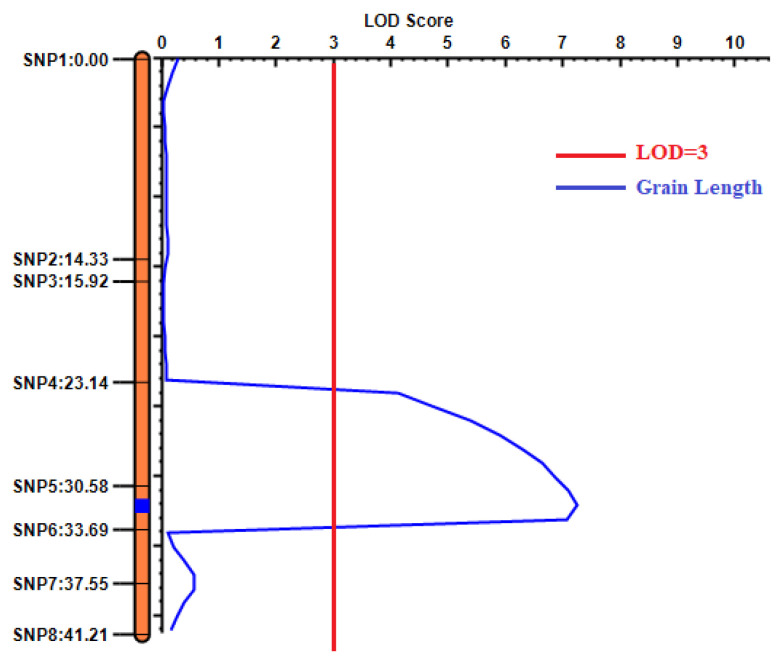
Linkage analysis of grain length.

For pyramid *qGL9.1*, eight KASP (kompetitive allele-specific PCR) markers were developed for linkage analysis based on the base information provided by re-sequencing data from Pin20, SJ15, and the two pools, and a significant peak interval was detected in a 448.7-kb region between SNP5 and SNP6 on chromosome 9 when the threshold was 3.0 (Figure 4). The *qGL9.1* contributed to 20.09% of the phenotypic variation for GL (Table 3). The positive-effect allele of *qGL9.1* was derived from Pin20. 

**Figure 5:**
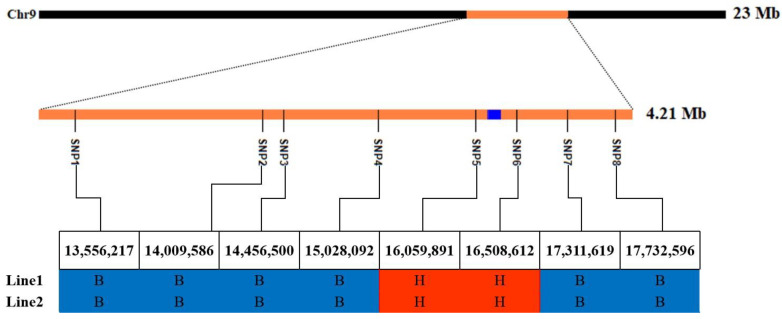
Screening of heterozygous lines in the BC_3_F_2_ population. Mb, million base pair; B, the genotype is consistent with SJ15; and H, the genotype is heterozygous.

**Figure 6:**
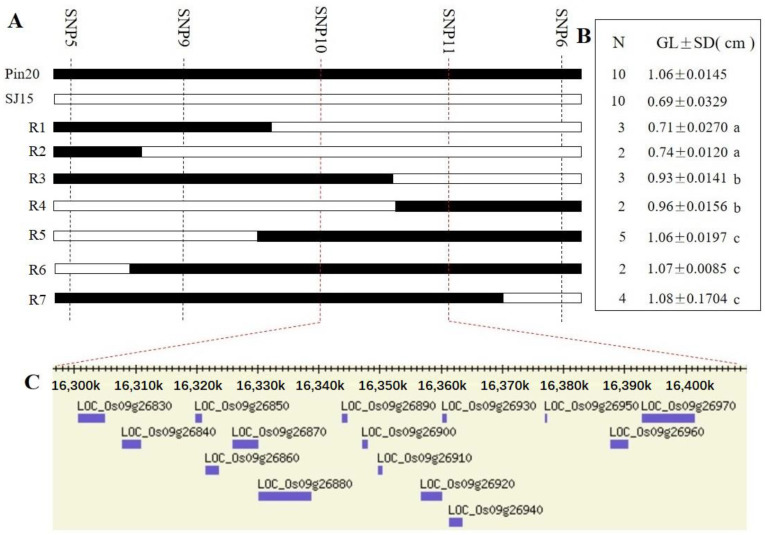
Fine mapping of *qGL9.1*. (**A**) The genotype of the recombinant plant. (**B**) Grain length statistics of recombinant plants. (**C**) A total of 15 genes in the *qGL9.1* region were obtained through the annotation information on the *Nipponbare* genome. Letters a, b and c indicate significant differences between groups, significant difference detected at *p* < 0.05 level.

In order to finely localize *qGL9.1*, we constructed the BC_3_F_2_ population and genotyped the BC_3_F_2_ population using the linkage markers of *qGL9.1* and six KASP markers consistent with the genetic background of SJ15 (Figure 5) and finally screened to two recombinants and obtained the BC_3_F_3_ population containing 1570 lines after self-crossing. For the fine mapping of *qGL9.1*, three KASP markers between SNP5 and SNP6 were developed from the re-sequencing data (Supplementary Table S2, Figure 6A). A total of 21 recombinants were identified by scanning the genotypes of 1570 BC_3_F_3_ individuals, and these 21 recombinants were classified into seven groups (Figure 6B). After progeny tests, the grain lengths of recombinant groups one and two were biased toward the short-grain parent SJ15, and the remaining recombinant groups were close to the long-grain parent Pin20. *qGL9.1* was delimited to the 93.0 Kb interval between the SNP10 and SNP11 markers (16.29–16.39 Mb). According to the MSU Rice Genome Annotation Project Release 7 [20], there are 15 protein-coding genes on the *qGL9.1* locus (Figure 6C), and information on SNP/InDel in the *qGL9.1* region (upstream, UTR3, downstream, and exonic) is listed in Supplementary Table S3. We found that 10 of the 15 genes had sequence differences in the promoter, exon, or downstream regions.

### 2.5. Candidate Gene Analysis

**Figure 7:**
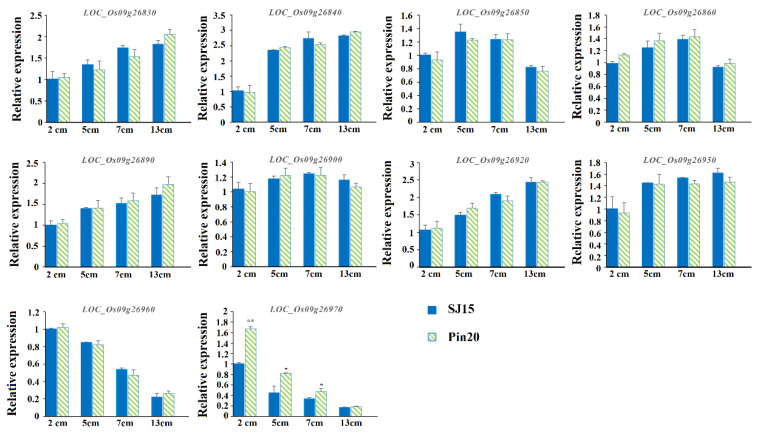
Expression of candidate genes during the development of the young panicles of both parents. The 2 cm, 5cm, 7cm, and 13 cm on the horizontal coordinates indicate the length of the developing young spike. The results were statistically analyzed using Student’s *t*-test (*, *p* < 0.05; **, *p* < 0.01).

**Figure 8:**
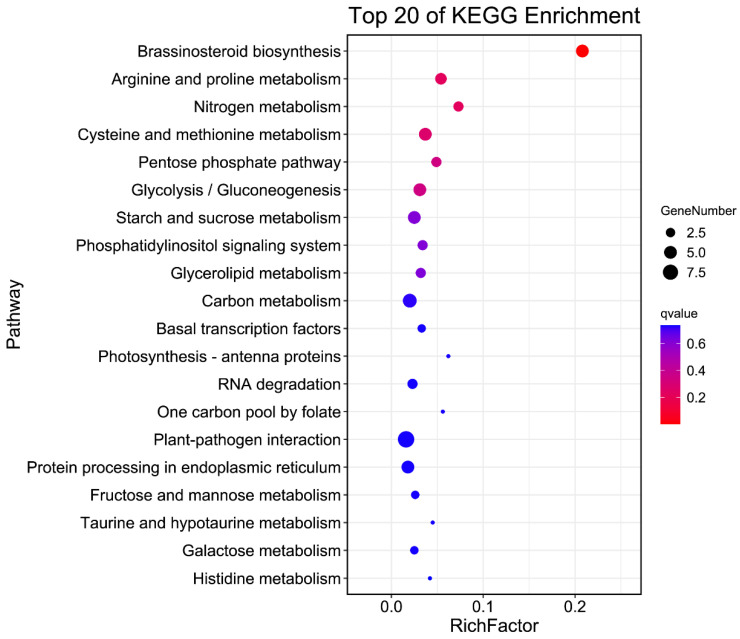
KO enrichment bubble plots for genes (Supplementary Table S5) in the *qGL9.1* interval. Horizontal coordinate: enrichment factor (number of differences in this pathway divided by all numbers); vertical coordinate: pathway name; bubble area size: number of genes belonging to this pathway in the target gene set; bubble color: enrichment significance. The redder the color, the smaller the P/Q value.

Through the qRT-PCR analysis of 10 genes with sequence variation (Figure 7), it was found that significant differences in the relative expression of *LOC_O09g26970* between Pin20 and SJ15 occurred in samples from the 2 cm, 5 cm, and 7 cm panicles, while no significant differences were found in the relative expression of the 13 cm panicles. The results showed that the relative expression of *LOC_Os09g26970* in Pin 20 was higher than that in SJ15 at the early stage of panicle development. The expression levels of the other nine genes were not significantly different at the grain development stage. We further analyzed the structural domains of 15 genes through the Pfam database, annotated them using the Ensembl database, and found that *LOC_Os09g26970* encodes a cytochrome P450 family protein CYP92A8 (Supplementary Table S4). In addition, using the results of gene annotation based on the re-sequencing data, through pathway significant enrichment analysis, it was found that 616 genes in the 4.2 Mb interval were significantly enriched in arginine and proline metabolism (ko00330), nitrogen metabolism (ko00910), cysteine and methionine metabolism (ko00270), pentose phosphate pathway (ko00030), and glycolysis/gluconeogenesis (ko00010) (Figure 8). Among them, five genes (*LOC_Os09g26940*, *LOC_Os09g26950*, *LOC_Os09g26960*, *LOC_Os09g26970,* and *LOC_Os09g26980*) in the candidate interval were significantly enriched in brassinolide biosynthesis (ko00905) (Supplementary Table S5). The genes encoding the cytochrome P450 family proteins have been shown to play an important role in regulating rice grain shape, especially *D11* [21], *GW10* [22], and other proteins encoding the cytochrome P450 family, which plays an active role in controlling grain size through the BR pathway. Therefore, as a P450 family protein significantly enriched in the BR pathway, we believe that *LOC_Os09g26970* is a candidate gene for *qGL9.1*.

### 2.6. The Significant Association of LOC_Os09g26970 with the SNP

Sanger sequencing analysis identified 13 nSNPs on *LOC_Os09g26970* (Supplementary Table S6). The 13 SNPs were previously identified in the 3010 Rice Genome Project and the Rice Functional and Genomic Breeding (RFGB) v2.0 database [23,24]. Among them, 10 SNPs (Chr9-16397163, Chr9-16397736, Chr9-16397760, Chr9-16397792, Chr9-16398197, Chr9-16398200, Chr9-16398479, Chr9-16398989, Chr9-16399274, and Chr9-16399673) constituted nine haplotypes, and Hap1, Hap5, Hap6, Hap8, and Hap9 were mainly distributed in *indica* rice. Hap2, Hap3, and Hap4 were mainly distributed in *japonica* rice (Supplementary Table S7). The germplasm of Hap9, consistent with the Pin 20 genotype, and the Hap2, consistent with the SJ15 genotype, differed significantly between GL and GW, and the other haplotypes caused significant phenotypic differences in GL, GW, and GL/W. (Supplementary Table S8). It is worth noting that the haplotype Hap9 contributes to the optimal GL (9.36 mm) and GL/W (3.31), suggesting that Pin20 is a cultivated species carrying the optimal grain length variation of *LOC_Os09g26970*. 

**Figure 9:**
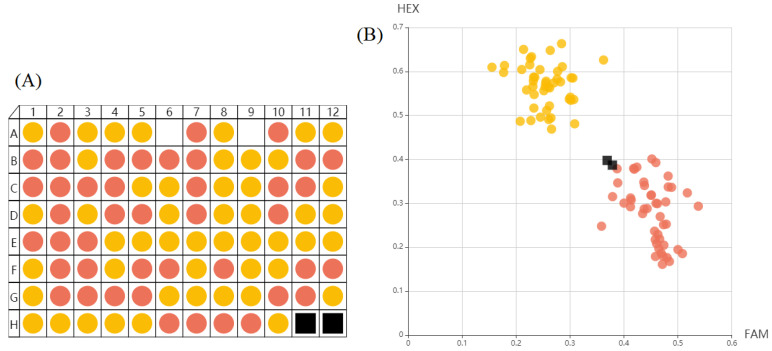
A total of 94 BC_2_F_4_ lines were genotyped with SNP10. (**A**) A demonstration of the genotyping effect of SNP10. Yellow fluorescence and red fluorescence indicate individuals with genotypes of Pin20 and SJ15, respectively. Black squares represent spotting holes without samples. The calls for the *LOC_Os09g26970-Pin20* allele were clustered near the *Y*-axis, while the calls for the *LOC_Os09g26970-SJ15* allele were clustered on the *X*-axis. (**B**) HEX, Hexachlorofluorescein; and FAM, 6-Carboxyfluorescein.

In order to obtain a molecular marker that could distinguish the grain length phenotype, we designed a KASP marker for an nSNP of the *LOC_Os09g26970*. SNP10 accurately divided the genotypes of 92 individuals in the 94 BC_3_F_3_ lines into Pin20 and SJ15 genotypes (Figure 9). These clustering results clearly distinguished the two alleles, therefore, the KASP8 marker was used to genotype the rice plants. Of the plants with the Pin20 allele, SNP10 identified 89.8% of the plants showing long-grain phenotypes. In contrast, SNP10 was able to identify 86.0% of the short-grain phenotype plants carrying the SJ15 genotype (Supplementary Table S9). This result implies that SNP10 can effectively distinguish the grain length of rice and can be used as an important molecular marker for breeding improvement.

## 3. Discussion

### 3.1. QTL-Seq Analysis Combined with a Screening of Recombinant Plants Can Efficiently Fine-Map Candidate Genes

Grain length is a significant factor that limits rice yield. Improving and utilizing the large-effect genomic loci associated with GL is essential to increase rice yield. The authors of previous studies have carried out extensive QTL analyses and localized a group of genes that are associated with GL in rice. For example, *PGL1* [25] and *BG1* [26] positively regulated GL by increasing the cell size, whereas *SG1* [27], *SDF5* [28], *OsGDI1* [29], and *TGW6* [8] negatively regulated rice GL by reducing the cell size. However, the strategy of isolating genes by map-based cloning is time-consuming and labor-intensive. In recent years, with the development and application of biological high-throughput sequencing technology and bioinformatics analysis technology, the efficiency of mining QTL has significantly improved. The combination of traditional QTL mapping and QTL-seq can effectively and quickly identify the GL major QTL interval. For example, the GL locus *qTGW5.3* was mapped to a 5 Mb physical interval by QTL-seq. Furthermore, the recombinants and the progeny tests delimited the candidate region of *qTGW5.3* to 1.13 Mb [30]. Due to the lack of further mapping populations and recombinant plants, the candidate genes of *qTGW5.3* have not been identified. In this study, *qGL9.1* was isolated from Pin20 by using the QTL-seq strategy based on the ED, Fisher algorithm, and G value method, and *qGL9.1* was associated with a single strong peak in the three calculation models (Figure 3). This shows a significant difference in the allele ratio between the two mixed pools. To fine map the *qGL9.1* candidate gene, several approaches have been used to narrow down the genomic region associated with *qGL9.1*. Firstly, *qGL9.1* was fine-mapped to a 93 Kb interval containing 15 annotated genes by using the recombinant plants to optimize the target interval (Figure 3). *LOC_Os09g26970* was further anchored as the most reliable candidate for *qGL9.1* by expression analysis and functional annotation of the candidate genes. Therefore, *qGL9.1* can be considered the most significant target for GL in exploring candidate genes. Our study is a good example of using QTL-seq combined with fine mapping to mine candidate genes to obtain major QTL intervals.

### 3.2. LOC_Os09g26970 on qGL9.1 Links to Grain Length in Rice

*LOC_O09g26970* is a cytochrome P450 structural domain (PF00067) gene, and the cytochrome P450 gene family is one of the largest supergene families in plants [31]. There are 356 P450 genes in the rice genome, and P450 plays an important role in various biochemical pathways that produce primary and secondary metabolites [32], some of which are essential for controlling plant cell proliferation and expansion. The proteins encoding cytochrome P450 families such as *D11* [21], *GW10* [22], *BSR2* [33], *GL3.2* [34], and *GE* [35] play an important role in regulating rice grain shape. In particular, the P450 family proteins encoded by *D11* and *GW10* play an active role in controlling the grain size through the biosynthetic pathway of brassinolide. In plants, BR is an essential steroid hormone that regulates many processes during plant development. It is involved in various biological reactions, such as stem elongation and vascular differentiation [36], especially in the regulation of grain size. Based on the re-sequencing data, this study found that *LOC_Os09g26970* was significantly enriched by KEGG enrichment analysis, which was related to the biosynthesis of brassinolide (ko00905). Therefore, it is speculated that the pathway of *qGL9.1* regulating grain shape is likely to be similar to that of *D11* and *GW10*. We further sequenced the CDS region of *LOC_Os09g26970* and found that there were 13 SNPs within it. Some of the haplotypes showed significant differences in grain length and grain width. We speculated that this locus was functional for grain length and grain width, but only showed differences in grain length in the genetic population of this study, which may have been caused by limited genetic variation. Next, we will construct various transgenic materials, such as knockout, overexpression, and complementation of *LOC_Os09g26970,* to verify its biological function in regulating rice grain length and analyze whether the effect of *LOC_Os09g26970* on grain length is affected by the BR pathway by applying exogenous BR.

### 3.3. Breeding Value and Potential of qGL9.1

In general, the cooking and eating quality of *japonica* rice is better than that of *indica* rice. While *indica* rice has longer grains and a better appearance quality than *japonica* rice, the quality of indica rice with long grains is often inferior to that of *japonica* rice [2,37]. In recent years, the molecular breeding and utilization of grain shape genes in *indica* and *japonica* rice completed several important tasks. New *indica* hybrid rice varieties, Taifengyou 55 and Taifengyou 208, with an improved grain yield and quality were developed by pyramiding semi-dominant *GS3* and *GW7^TFA^* alleles from tropical *japonica* rice varieties [38]. The *GW8* and *GS3* alleles were polymerized into HJX74 to produce short and wide grains, resulting in the breeding of Huabiao 1 [12]. Using the deletion of *TGW6* and its alleles in the functional region, the functional marker CAPs6-1 of *TGW6* was developed and screened in order to quickly screen rice varieties carrying *TGW6* [39]. In this study, allelic variation A from *japonica* Pin20 was present in a small number of indica rice samples, but has not been identified in other *japonica* rice samples, indicating that *LOC_Os09g26970* may be a rare grain shape regulator in *japonica* rice germplasm. In addition, 10 SNPs in the coding region of *LOC_Os09g26970* had nine haplotypes in 3010 rice germplasms. The grain length of the germplasm containing the Pin20 genotype was 9.36 mm, while the average grain length of the other haplotypes was 8.60 mm. Therefore, Hap9 is the optimal haplotype of grain length, and Hap9 has the largest GL/W, which is an ideal allelic variation related to grain length. In addition, the GL/W of the germplasm corresponding to the nine haplotypes showed long-grain characteristics (the minimum GL/W was 2.52). Therefore, the nine haplotypes of *LOC_O09g26970* are helpful to determine the grain length of rice germplasm. Moreover, we selected one SNP from Hap9 as a molecular marker to analyze the individuals with significant differences in grain length in the BC_3_F_3_ population and found that SNP10 could be used as a target for marker grain length. Next, in *India–japonica* hybrid breeding, the selection of Hap9 can not only retain the excellent quality traits of japonica rice, but also help to improve its grain length. The KASP marker used in the study is a marker that is closely linked to GL, and it can be directly used for molecular-assisted selection. In addition, this locus can be inherited by offspring by inter-*japonica* hybridization, and long-grain varieties can be directly selected by conventional breeding methods.

## 4. Materials and Methods

### 4.1. Plant Materials

In this study, two japonica varieties, short-grain female parent SJ15 and long-grain male parent Pin20, were used as parental lines to develop 667 F_2_ individuals and the corresponding F_2:3_ population. The F_2_ population was planted during the normal growing season (from April to October) and the mature seeds were harvested subsequently. All 667 F_2:3_ lines were used for grain type identification after maturity. To fine map the target gene, one F_2_ individual plant with long grains was selected to obtain BC_3_F_1_ seeds by backcrossing with SJ15, and a BC_3_F_1_ individual plant with a long-grain phenotype was self-crossed to generate the BC_3_F_2_ (725 individuals) and BC_3_F_3_ (1570 individuals) populations. The 667 F_2:3_ individuals were used for QTL-seq and QTL mapping, and BC_3_F_2_ and BC_3_F_3_ were used to fine-map the *qGL9.1* candidate gene. All of the lines and their parents were planted at the Northeast Agricultural University experimental station (Heilongjiang Province, China; 47°98 N, 128°08 E; 128 m above sea level). 

### 4.2. Evaluation of Grain Type for Rice

The grain size of the F_2:3_ and BC_3_F_3_ populations was investigated when the rice was fully mature. We collected all of the spikes of each line in envelopes, placed them in natural light to dry, and then put them in an oven at 37 °C for one week. Three main spikes of each line, with approximately the same appearance, were randomly selected and used to measure the spike length and spike grain number. The grain length (GL) and grain width (GW) of 10 seeds of each line were measured with vernier calipers and the ratio of GL to GW was calculated. The phenotypic data for each line were measured in three replicates, and their average was used for data statistics.

### 4.3. Construction of Segregating Pools and Whole-Genome Re-Sequencing

Young leaves from 667 individuals of the F_2_ population were collected separately for total genomic DNA extraction using a modified cetyltrimethylammonium bromide (CTAB) method [40]. Then, the genomic DNA of 30 extremely GL-type and 30 extremely GW-type individuals were selected as two bulked pools. To simplify the following description, we abbreviated the GL-type DNA pool as GL-pool, and the GW-type DNA pool as GW-pool. For GL-pool, GW-pool, and the two parents, isolated DNA was quantified using a Nanodrop 2000 spectrophotometer (Thermo Scientific, Fremont, CA, USA). All DNA from the GL-pool and GW-pool was quantified at precise concentrations with a Qubit^®^ 2.0 Fluorometer (Life Technologies, Carlsbad, CA, USA). Equal amounts of DNA from the GL-pool and GW-pool plants were mixed. The four DNA libraries were sequenced on the Illumina MiSeq platform using the MiSeq Reagent Kit v2 (500 cycles) (Illumina Inc., San Diego, CA, USA). 

### 4.4. QTL-Seq Analysis

The raw sequencing data were filtered using an internal Perl script, provided by Biomarker Technology Co. Ltd. (Beijing, China). These high-quality data were then mapped to the Nipponbare-Reference-IRGSP-1.0 [41] using the Burrows–Wheeler aligner [42]. Using the Picard tool (https://sourceforge.net/projects/picard/) (accessed on 8 June 2021), repeat reads were removed based on the clean reads located in the reference genome. The SNP and InDel (1–5 bp) calling was realized with GATK [43], using the default settings. A series of filters were also used to obtain highly accurate SNP and InDel sets [44]. The association analysis was performed using the ED [45], calculation of the G statistic [46,47], and two-tailed Fisher’s exact test [48] based on SNP. Finally, the overlapping interval of the three methods was used as the QTL interval. 

### 4.5. Further Mapping of the qGL9.1

To further delimit the position of *qGL9.1*, we developed KASP markers linked to the *qGL9.1* interval, and then KASP marker primers were designed with Primer 5 software (Premier Biosoft International, Corina Way, Palo Alto, CA, USA) based on the re-sequencing data of the two parents. The 5′ end of each KASP marker forward primer was ligated with FAM (5′-GAAGGTGACCAAGTTCATGCT-3′) and HEX (5′-GAAGGTCGGAGTCAACGGATT-3′) linker sequences. All polymorphic markers between the parents were selected and 667 F_2_ individuals were genotyped using polymorphic markers to construct linkage maps and narrow down the candidate regions using the inclusive composite interval mapping (ICIM) module of QTL IciMapping 4.2. (http://www.isbreeding.net) (accessed on 19 June 2023) and are listed in Supplementary Table S2. The threshold of the LOD score for declaring the presence of a significant QTL was determined by a permutation test with 1000 repetitions at *p* < 0.001. Then, 725 BC_3_F_2_ individuals and 1570 BC_3_F_3_ individuals were used to screen the recombinants across the kompetitive allele-specific PCR (KASP) markers between the target regions. Each KASP marker contained two allele-specific forward primers and one common reverse primer. The reaction mixture was prepared according to the protocol described by KBiosciences (http://www.ksre.ksu.edu/igenomics) (accessed on 19 June 2023). All of the KASP primers are listed in Supplementary Table S2. 

### 4.6. Fine Mapping and Candidate Gene Screening of qGL9.1

To fine map *qGL9.1*, the plants with interval heterozygous *qGL9.1* in the BC_3_F_2_ population were identified by the KASP marker, and the BC_3_F_3_ secondary population was obtained by selfing. The BC_3_F_3_ population was genotyped, and the recombinant plants were screened to achieve fine-mapped *qGL9.1*. The main methods of mining candidate genes were as follows: (1) Ensembl (http://ensemblgenomes.org/) (accessed on 19 June 2023) was used to annotate the candidate genes, and the possible domains of candidate genes were detected by the Pfam database (http://pfam.xfam.org/) (accessed on 19 June 2023). (2) Mutant genes were screened according to sequencing information. (3) The genes with sequence variation were analyzed by using qRT-PCR. When the young panicles began to differentiate, those of Pin 20 and SJ15 were sampled at the lengths of 2cm, 5cm, 7cm, and 13cm. The expression characteristics of the candidate genes between the parents were analyzed by using qRT-PCR.

The total RNA of the rice was extracted according to the steps of the GeneCopoeia-BlazeTaq™ SYBR^®^ Green qPCR Mix 2.0 extraction kit, and RNA purification and reverse transcription were carried out according to the steps of SIMGEN of Hangzhou Xinjing Biological Reagent Co., Ltd (No.8, Xiyuan 1st Road, Xihu District, Hangzhou, Zhejiang, China). Amplification was performed with a Roche LightCycler96 fluorescence quantitative PCR instrument at Northeastern Agricultural University. According to the transcription sequence of the gene, the specific primers of the candidate gene were designed with Premier 5.0 software, and the sequence is shown in Supplementary Table S10. The original *Actin1* in the rice was used as the internal reference [49], and the specificity of the primers was based on the standard melting curve. Three replicates were set for each sample, and the relative expression of genes in tissues was calculated using the the 2-ΔΔCt method. qRT-PCR analysis was performed as previously described [50].

### 4.7. Haplotype Analysis of Candidate Genes

According to the RFGB database (Haplotype analysis module of https://www.rmbreeding.cn/index.php (accessed on 19 June 2023)), the differential bases in the coding region of candidate genes between parents were searched, and the haplotypes of these differential bases in 3010 rice varieties and the variation of each haplotype in the different rice germplasms were analyzed. The phenotypic data of the grain length, grain width, and aspect ratio in the RFGB database and their genomic information were used to analyze the differences between the different haplotypes of the candidate genes. 

### 4.8. Development of KASP Markers and Validation of GL

To verify the above-identified *LOC_Os09g26970* with GL potential, two non-synonymous SNPs (nSNPs) were screened from the exons of *LOC_Os09g26970*, and the corresponding KASP markers were developed. The upstream and downstream 100-bp sequences of the target nSNPs were extracted from the Nipponbare genome sequence. Each KASP marker contained two allele-specific forward primers and a common reverse primer. The reaction mixture was prepared according to the instructions of KBiosciences (http://www.ksre.ksu.edu/igenomics (accessed on 19 June 2023)), and the KASP primers are shown in Supplementary Table S2. 

## 5. Conclusions

In this study, we used F_2_ and BC_3_F_3_ populations to identify a major QTL *qGL9.1* controlling rice grain length from long-grain variety Pin20 by re-sequencing and fine mapping. Furthermore, combined with functional annotation, variation detection, and qRT-PCR analysis, the gene *LOC_Os09g26970* encoding a P450 protein was identified as a candidate gene for *qGL9.1*. *LOC_Os09g26970* and was divided into nine haplotypes in 3010 rice germplasm, and Hap9, which was consistent with the genotype of Pin20, contributed the most to the grain length among all of the haplotypes. In summary, we found a new grain length gene in early-maturing *japonica* rice in the northernmost part of China, and the molecular breeding application of this gene will hopefully assist in tackling the difficult situation of improving the yield of early-maturing *japonica* rice.

## Figures and Tables

**Table 1:** Phenotypic analysis of grain-shape-related traits of parents and populations.

Traits	SJ15	Pin20	F_2:3_ Population			
			Mean ± SD	Range	Skewness	Kurtosis
GL (cm)	0.69	1.06 **	0.85 ± 0.00	0.68~1.09	0.61	0.95
GW (cm)	0.36	0.40	0.37 ± 0.00	0.33~0.43	0.27	1.46
GL/W	1.92	2.65 **	2.55 ± 0.02	1.88~3.09	0.33	−0.05

** indicates the significant difference detected at *p* < 0.01 level.

**Table 2:** *qGL9.1* association results based on the G-statistic value, ED algorithm, and Fisher algorithm.

QTL	Algorithm	Start (bp)	End (bp)	Size (Mb)	Gene Number	Threshold
*qGL9.1*	G-statistic	14,185,459	18,445,701	4.26	623	0.01
	ED	14,240,001	18,760,000	4.52	669	0.01
	Fisher algorithm	13,560,001	19,030,000	5.47	807	0.05

**Table 3:** QTL association results by linkage analysis.

Trait	Chr.	Position (cM)	LOD	PVE (%)	Add	Left CI (cM)	Right CI (cM)
GL	9	32.00	7.23	20.09	0.03	27.5	33.5

Note: GL, grain length; Chr., chromosome; cM, centimorgan; LOD, logarithm of the maximum likelihood; PVE, phenotypic variation explained; Add, additive effect; Left CI, confidence interval on the left side of the linkage map; and Rigth CI, confidence interval on the right side of the linkage map.

## Data Availability

The data presented in this study are available on request from the corresponding author. The data are not publicly available due to much unpublished genomic information from the resequencing data.

## Supplementary Materials

The following supporting information can be downloaded at: https://www.mdpi.com/article/10.3390/ijms241411447/s1.

## References

[1] Sakamoto T., Matsuoka M. (2008). Identifying and exploiting grain yield genes in rice. Curr. Opin. Plant Biol..

[2] Harberd N.P. (2015). Shaping Taste: The Molecular Discovery of Rice Genes Improving Grain Size, Shape and Quality. J. Genet. Genom. Yi Chuan Xue Bao.

[3] Ren D., Ding C., Qian Q. (2023). Molecular bases of rice grain size and quality for optimized productivity. Sci. Bull..

[4] Choi B.S., Kim Y.J., Markkandan K., Koo Y.J., Song J.T., Seo H.S. (2018). *GW2* Functions as an E3 Ubiquitin Ligase for Rice Expansin-Like 1. Int. J. Mol. Sci..

[5] Utsunomiya Y., Samejima C., Takayanagi Y., Izawa Y., Yoshida T., Sawada Y., Fujisawa Y., Kato H., Iwasaki Y. (2011). Suppression of the rice heterotrimeric G protein β-subunit gene, RGB1, causes dwarfism and browning of internodes and lamina joint regions. Plant J..

[6] Kunihiro S., Saito T., Matsuda T., Inoue M., Kuramata M., Taguchi-Shiobara F., Youssefian S., Berberich T., Kusano T. (2013). Rice DEP1, encoding a highly cysteine-rich G protein γ subunit, confers cadmium tolerance on yeast cells and plants. J. Exp. Bot..

[7] Abu-Zaitoon Y.M., Bennett K., Normanly J., Nonhebel H.M. (2012). A large increase in IAA during development of rice grains correlates with the expression of tryptophan aminotransferase *OsTAR1* and a grain-specific YUCCA. Physiol. Plant.

[8] Ishimaru K., Hirotsu N., Madoka Y., Murakami N., Hara N., Onodera H., Kashiwagi T., Ujiie K., Shimizu B., Onishi A. (2013). Loss of function of the IAA-glucose hydrolase gene *TGW6* enhances rice grain weight and increases yield. Nat. Genet..

[9] Duan P., Ni S., Wang J., Zhang B., Xu R., Wang Y., Chen H., Zhu X., Li Y. (2015). Regulation of *OsGRF4* by *OsmiR396* controls grain size and yield in rice. Nat. Plants.

[10] Schmidt R., Schippers J.H., Mieulet D., Watanabe M., Hoefgen R., Guiderdoni E., Mueller-Roeber B. (2014). SALT-RESPONSIVE ERF1 is a negative regulator of grain filling and gibberellin-mediated seedling establishment in rice. Mol. Plant.

[11] Takano-Kai N., Doi K., Yoshimura A. (2011). *GS3* participates in stigma exsertion as well as seed length in rice. Breed. Sci..

[12] Wang S., Wu K., Yuan Q., Liu X., Liu Z., Lin X., Zeng R., Zhu H., Dong G., Qian Q. (2012). Control of grain size, shape and quality by OsSPL16 in rice. Nat. Genet..

[13] Yu J., Xiong H., Zhu X., Zhang H., Li H., Miao J., Wang W., Tang Z., Zhang Z., Yao G. (2017). *OsLG3* contributing to rice grain length and yield was mined by Ho-LAMap. BMC Biol..

[14] Si L., Chen J., Huang X., Gong H., Luo J., Hou Q., Zhou T., Lu T., Zhu J., Shangguan Y. (2016). *OsSPL13* controls grain size in cultivated rice. Nat. Genet..

[15] Xu Y., Wang R., Wang Y., Zhang L., Yao S. (2020). A point mutation in *LTT1* enhances cold tolerance at the booting stage in rice. Plant Cell Environ..

[16] Kodama A., Narita R., Yamaguchi M., Hisano H., Adachi S., Takagi H., Ookawa T., Sato K., Hirasawa T. (2018). QTLs maintaining grain fertility under salt stress detected by exome QTL-seq and interval mapping in barley. Breed. Sci..

[17] Tiwari S., Sl K., Kumar V., Singh B., Rao A.R., Mithra Sv A., Rai V., Singh A.K., Singh N.K. (2016). Mapping QTLs for Salt Tolerance in Rice (*Oryza sativa* L.) by Bulked Segregant Analysis of Recombinant Inbred Lines Using 50K SNP Chip. PLoS ONE.

[18] Wu F., Yang J., Yu D., Xu P. (2020). Identification and validation a major QTL from “Sea Rice 86” seedlings conferred salt tolerance. Agronomy.

[19] Shamaya N.J., Shavrukov Y., Langridge P., Roy S.J., Tester M. (2017). Genetics of Na(+) exclusion and salinity tolerance in Afghani durum wheat landraces. BMC Plant Biol..

[20] Kawahara Y., de la Bastide M., Hamilton J.P., Kanamori H., McCombie W.R., Ouyang S., Schwartz D.C., Tanaka T., Wu J., Zhou S. (2013). Improvement of the *Oryza sativa* Nipponbare reference genome using next generation sequence and optical map data. Rice.

[21] Zhu X., Liang W., Cui X., Chen M., Yin C., Luo Z., Zhu J., Lucas W.J., Wang Z., Zhang D. (2015). Brassinosteroids promote development of rice pollen grains and seeds by triggering expression of Carbon Starved Anther, a MYB domain protein. Plant J..

[22] Zhan P., Wei X., Xiao Z., Wang X., Ma S., Lin S., Li F., Bu S., Liu Z., Zhu H. (2021). GW10, a member of P450 subfamily regulates grain size and grain number in rice. Theor. Appl. Genet..

[23] Wang C.C., Yu H., Huang J., Wang W.S., Faruquee M., Zhang F., Zhao X.Q., Fu B.Y., Chen K., Zhang H.L. (2020). Towards a deeper haplotype mining of complex traits in rice with RFGB v2.0. Plant Biotechnol. J..

[24] Wang W., Mauleon R., Hu Z., Chebotarov D., Tai S., Wu Z., Li M., Zheng T., Fuentes R.R., Zhang F. (2018). Genomic variation in 3010 diverse accessions of Asian cultivated rice. Nature.

[25] Heang D., Sassa H. (2012). Antagonistic actions of HLH/bHLH proteins are involved in grain length and weight in rice. PLoS ONE.

[26] Liu L., Tong H., Xiao Y., Che R., Xu F., Hu B., Liang C., Chu J., Li J., Chu C. (2015). Activation of Big Grain1 significantly improves grain size by regulating auxin transport in rice. Proc. Natl. Acad. Sci. USA.

[27] Nakagawa H., Tanaka A., Tanabata T., Ohtake M., Fujioka S., Nakamura H., Ichikawa H., Mori M. (2012). Short grain1 decreases organ elongation and brassinosteroid response in rice. Plant Physiol..

[28] Yang Y., Li J., Li H., Xu Z., Qin R., Wu W., Wei P., Ding Y., Yang J. (2021). SDF5 Encoding P450 Protein Is Required for Internode Elongation in Rice. Rice Sci..

[29] Ali Shad M., Wang Y., Zhang H., Zhai S., Shalmani A., Li Y. (2023). Genetic analysis of GEFs and GDIs in rice reveals the roles of OsGEF5, OsGDI1, and OsGEF3 in the regulation of grain size and plant height. Crop J..

[30] Qin Y., Cheng P., Cheng Y., Feng Y., Huang D., Huang T., Song X., Ying J. (2018). QTL-Seq Identified a Major QTL for Grain Length and Weight in Rice Using Near Isogenic F_2 Population. Rice Sci..

[31] Nelson D.R. (1998). Cytochrome P450 nomenclature. Methods Mol. Biol. (Clifton N.J.).

[32] Schuler M.A., Werck-Reichhart D. (2003). Functional genomics of P450s. Annu. Rev. Plant Biol..

[33] Maeda S., Sasaki K., Kaku H., Kanda Y., Ohtsubo N., Mori M. (2022). Overexpression of Rice BSR2 Confers Disease Resistance and Induces Enlarged Flowers in Torenia fournieri Lind. Int. J. Mol. Sci..

[34] Xu F., Fang J., Ou S., Gao S., Zhang F., Du L., Xiao Y., Wang H., Sun X., Chu J. (2015). Variations in CYP78A13 coding region influence grain size and yield in rice. Plant Cell Environ..

[35] Nagasawa N., Hibara K., Heppard E.P., Vander Velden K.A., Luck S., Beatty M., Nagato Y., Sakai H. (2013). GIANT EMBRYO encodes CYP78A13, required for proper size balance between embryo and endosperm in rice. Plant J..

[36] Abe Y., Mieda K., Ando T., Kono I., Yano M., Kitano H., Iwasaki Y. (2010). The SMALL AND ROUND SEED1 (SRS1/DEP2) gene is involved in the regulation of seed size in rice. Genes Genet. Syst..

[37] Hirose T., Aoki N., Harada Y., Okamura M., Hashida Y., Ohsugi R., Akio M., Hirochika H., Terao T. (2013). Disruption of a rice gene for α-glucan water dikinase, *OsGWD1*, leads to hyperaccumulation of starch in leaves but exhibits limited effects on growth. Front. Plant Sci..

[38] Wang S., Li S., Liu Q., Wu K., Zhang J., Wang S., Wang Y., Chen X., Zhang Y., Gao C. (2015). The *OsSPL16-GW7* regulatory module determines grain shape and simultaneously improves rice yield and grain quality. Nat. Genet..

[39] Wang J., Yang J., Xu X., Zhu J., Fan F., Li W., Wang F., Zhong W. (2014). Development and Application of a Functional Marker for Grain Weight Gene TGW6 in Rice. Chin. J. Rice Sci..

[40] Murray M.G., Thompson W.F. (1980). Rapid isolation of high molecular weight plant DNA. Nucleic Acids Res..

[41] Sasaki T. (2005). The map-based sequence of the rice genome. Nature.

[42] Li H., Durbin R. (2009). Fast and accurate short read alignment with Burrows–Wheeler transform. Bioinformatics.

[43] McKenna A., Hanna M., Banks E., Sivachenko A., Cibulskis K., Kernytsky A., Garimella K., Altshuler D., Gabriel S., Daly M. (2010). The Genome Analysis Toolkit: A MapReduce framework for analyzing next-generation DNA sequencing data. Genome Res..

[44] Reumers J., De Rijk P., Zhao H., Liekens A., Smeets D., Cleary J., Van Loo P., Van Den Bossche M., Catthoor K., Sabbe B. (2011). Optimized filtering reduces the error rate in detecting genomic variants by short-read sequencing. Nat. Biotechnol..

[45] Hill J.T., Demarest B.L., Bisgrove B.W., Gorsi B., Su Y.C., Yost H.J. (2013). MMAPPR: Mutation mapping analysis pipeline for pooled RNA-seq. Genome Res..

[46] Magwene P.M., Willis J.H., Kelly J.K. (2011). The Statistics of Bulk Segregant Analysis Using Next Generation Sequencing. PLoS Comp. Biol..

[47] Mansfeld B.N., Grumet R. (2018). QTLseqr: An R Package for Bulk Segregant Analysis with Next-Generation Sequencing. Plant Genome.

[48] Fisher R.A. (1922). On the interpretation of χ^2^ from contingency tables, and the calculation of P. J. R. Stat. Soc..

[49] Siahpoosh M.R., Sanchez D.H., Schlereth A., Scofield G.N., Furbank R.T., van Dongen J.T., Kopka J. (2012). Modification of *OsSUT1* gene expression modulates the salt response of rice Oryza sativa cv. Taipei 309. Plant Sci..

[50] Zhang Z.Y., Li J.J., Tang Z.S., Sun X.M., Zhang H.L., Yu J.P., Yao G.X., Li G.L., Guo H.F., Li J.L. (2018). Gnp4/LAX2, a RAWUL protein, interferes with the *OsIAA3-OsARF25* interaction to regulate grain length via the auxin signaling pathway in rice. J. Exp. Bot..

